# Decoding ruminative reflection in healthy individuals: The role of triple network connectivity

**DOI:** 10.1016/j.ijchp.2024.100508

**Published:** 2024-10-10

**Authors:** Luqing Wei, Hui Dong, Zijing Zhang, Chris Baeken, Yige Wang, Guo-Rong Wu

**Affiliations:** a Key Laboratory of Cognition and Personality, Faculty of Psychology, Southwest University, Chongqing, China; b School of Psychology, Jiangxi Normal University, Nanchang, China; c Ghent Experimental Psychiatry Lab, Department of Head and Skin, UZ Gent/Universiteit Gent, Ghent, Belgium; d Department of Psychiatry, Center for Neurosciences (C4N), UZ Brussel/ Neuroprotection and Neuromodulation Research Group (NEUR), Vrije Universiteit Brussel (VUB), Brussels, Belgium; e Department of Electrical Engineering, Eindhoven University of Technology, Eindhoven, the Netherlands; f MOE Key Laboratory for Neuroinformation, University of Electronic Science and Technology of China, Chengdu, China

**Keywords:** Reflection, Executive control, Functional connectivity, Triple-network model

## Abstract

Ruminative reflection has been linked to enhanced executive control in processing internally represented emotional information, suggesting it may serve as an adaptive strategy for emotion regulation. Investigating the neural substrates of reflection can deepen our understanding of its adaptive properties. This study used network-based statistic (NBS)-Predict methodology to identify resting state functional connectivity (FC)-based predictors of ruminative reflection in a healthy sample. Our results showed that reflection in healthy subjects was predicted by FC within and between the default mode network (DMN), fronto-parietal network (FPN), and salience network (SN). Notably, FC within the FPN and SN, as well as between the FPN and DMN, contributed more significantly to the predictive model. These results underscore the greater influence of FPN and SN connectivity in predicting reflection, providing empirical evidence that increased executive control over internal emotional representations is integral to adaptive reflective processes. Moreover, the triple-network model, particularly the FPN-DMN coupling, emerges as a crucial predictor of ruminative reflection, highlighting the importance of coordinating self-relevant and goal-directed processing in reflective mechanisms. These identified connectivity fingerprints may offer insights into the role of reflective processes in facilitating recovery from depression.

## Introduction

Rumination, characterized by repetitive thoughts centered on negative internal states, is a multidimensional construct comprising two components: ruminative brooding and ruminative reflection (Nolen-Hoeksema et al., 2008; Treynor et al., 2003). Ruminative reflection is a purposeful, self-distanced cognitive process aimed at understanding distress and emotions to prepare for active coping, whereas brooding is a passive, self-absorbed cognitive process that immerses individuals in negative emotions, often impeding disengagement (Satyshur et al., 2018). Previous studies have shown that these two subtypes of rumination exert divergent influences on the development of depressive symptoms (Burwell & Shirk, 2007; Cox et al., 2012; Hasegawa et al., 2013; Pearson et al., 2010; Schoofs et al., 2010; Treynor et al., 2003). For example, brooding correlates with depressive symptoms both concurrently and longitudinally (Burwell & Shirk, 2007; Treynor et al., 2003). Conversely, reflection, when accounting for brooding, shows no significant correlation with depressive symptoms and may facilitate goal clarification within the problem-solving frameworks (Burwell & Shirk, 2007). Additionally, brooding and reflection have different impacts on both positive and negative outcomes. Reflection is associated with improved executive control and enhanced creative behavior, whereas brooding is primarily linked to depressive mood (e.g., dysphoria) (Stewart et al., 2019; Verhaeghen et al., 2014). Brooding, but not reflection, consistently correlates with negative cognitive functioning, including negative attentional biases (Duque et al., 2014; Owens & Gibb, 2017), unhealthy perfectionism (Kun et al., 2020; Olson & Kwon, 2008), and passive coping responses (Burwell & Shirk, 2007; Marroquín et al., 2010). These findings collectively indicate that brooding and reflection may represent maladaptive and more adaptive (or less maladaptive) aspects of rumination, respectively.

Neuroimaging studies exploring the neural substrates of rumination have revealed distinct associations for brooding and reflection in healthy and/or depressed samples (Berman et al., 2011; Ehrlich et al., 2022; Hamilton et al., 2011; Li et al., 2022; Luo et al., 2016; Ordaz et al., 2017; Satyshur et al., 2018), emphasizing the necessity of examining these two components separately. For instance, brooding is related to functional connectivity (FC) between the insula and the parahippocampal gyrus/hippocampus (Li et al., 2022), as well as between the amygdala and the temporal pole (Lydon-Staley et al., 2019), highlighting the role of the salience network (SN). Additionally, FC within the default mode network (DMN), particularly involving the medial prefrontal cortex (MPFC) and posterior cingulate cortex (PCC), further supports the brooding construct (Berman et al., 2011; Luo et al., 2016). The interplay among the DMN, SN, and fronto-parietal network (FPN)- collectively known as the triple-network model-have also been implicated in ruminative brooding (Hamilton et al., 2011; Pisner et al., 2019). While the neural mechanisms underlying brooding have been extensively studied due to its association with depressive vulnerability, reflection remains comparatively underexplored. As previously noted, reflection is an active introspective process aimed at understanding one's own thoughts, feelings, and actions (Treynor et al., 2003). This reflective engagement enhances the ability to evaluate and refine task-orientated coping and problem-solving strategies, ultimately promoting psychological adjustment (Crane et al., 2019; Takano & Tanno, 2009). Emerging evidence suggests that reflection is associated with increased executive control during the processing of internally represented emotional information (Stewart et al., 2019), serving as a potential strategy for regulating negative emotions (Cristea et al., 2013) and strengthening resilience (Bucknell et al., 2022; Crane et al., 2019). Moreover, reflection is considered an active problem-solving cognitive process that predicts subsequent reductions in depression severity (Ehrlich et al., 2022; Mori et al., 2015). These findings point to reflection as a potentially adaptive form of rumination with important clinical implications (Stewart et al., 2019). Further research into the neural mechanisms underlying reflective rumination could enrich our knowledge of its adaptive nature.

The current study aimed to identify resting state FC-based predictors of reflection in a healthy sample by using the network-based statistics (NBS)-Predict methodology (Serin et al., 2021). NBS-Predict is a prediction-based extension of the NBS (Zalesky et al., 2010), integrating machine learning and graph theory in a cross-validation (CV) framework. This approach offers a fast and user-friendly tool for identifying neuroimaging biomarkers with high generalizability. Previous research indicates that brooding and reflection tend to exacerbate each other in depressed individuals, complicating their differentiation (Joormann et al., 2006). In contrast, these constructs can be more clearly distinguished in healthy individuals (Whitmer & Gotlib, 2011). Exploring the neural mechanisms of reflection in healthy cohort is crucial for comprehending its compensatory role in depression recovery (Ehrlich et al., 2022). Given that reflection is linked to FC between the insula subregion and the dorsolateral prefrontal cortex (DLPFC), anterior cingulate cortex (ACC), and PCC, as well as between the lateral orbitofrontal cortex and the MPFC/PCC (Ehrlich et al., 2022; Li et al., 2022; Satyshur et al., 2018), we hypothesize that functional interactions between the DMN, FPN, and SN would predict reflective rumination. Furthermore, reflection is associated with enhanced executive control over internal emotional representation and functions as a coping strategy (Cristea et al., 2013; Stewart et al., 2019). Thus, we expected that FC within cognitive control-related networks, specifically the FPN and SN, would significantly contribute to predicting reflection.

## Methods and materials

### Participants

A total of 1494 participants were initially recruited from the publicly available Enhanced Nathan Kline Institute-Rockland Sample (NKI-RS). All participants underwent multimodal MRI scans, semi-structured diagnostic psychiatric interviews, and a battery of psychiatric, cognitive, and behavioral assessments (Nooner et al., 2012). From this pool, 365 individuals were selected based on the availability of complete demographic data and Ruminative Responses Scale (RRS) scores. For the present study, additional screening was conducted to exclude individuals with any current medical conditions, as well as those with a history of mental health or substance use disorders. This process yielded a final sample of 84 healthy participants (18 males, 66 females), aged between 39 and 72 years (*M* = 54.48, SD=9.085). A flowchart illustrating exclusion criteria and participant selection is provided in the supplementary Fig. S1. The study protocols were reviewed and approved by the ethics committees of the NKI institutions. Informed consent was obtained from all subjects in accordance with the Declaration of Helsinki.

### Behavioral assessments

This study focused on two behavioral assessments: the Beck Depression Inventory (BDI) and the Ruminative Responses Scale (RRS). The BDI-II is a widely used self-report inventory that assessed the severity of depressive symptoms experienced over the preceding two weeks (Beck et al., 1996). The RRS consists of three subscales (i.e., Brooding, Reflection, and Depression) and 22 items (Treynor et al., 2003). Each item is rated on a 4-points scale (1= never to 4 = always), reflecting the extent to which individuals engage in ruminative response when feeling low. The RRS is a valid and reliable measure of rumination in both healthy and depressed individuals (Luminet, 2003). The demographic information and behavioral ratings are presented in Table 1.

**Table 1:** Participant demographic and questionnaire descriptive statistics.

Variables	Mean (Standard deviation)
Age	54.48 (9.085)
Female: Male	66:18
RRS-Brooding	8.21 (2.62)
RRS-Reflection	7.23 (2.55)
RRS-Total	33.77 (10.39)
BDI-II	5.54 (7.19)

Note: RRS, Ruminative Responses Scale; BDI-II, Beck Depression Inventory-II.

### Data acquisition and preprocessing

Image data were acquired using a Siemens Tim Trio 3T scanner. Resting-state functional images were acquired using a multiband echo-planar imaging (EPI) sequence with the following settings: TR = 0.645 s, TE = 0.03 s, FA = 60°, 40 slices, resolution = 3 mm × 3 mm × 3 mm. A total of 900 vol were acquired for each subject, with participants instructed to keep their eyes fixated on a crosshair, stay still, and not think of anything in particular. Anatomical images were recorded using a magnetization-prepared rapid gradient-echo (MPRAGE) sequence (TR/TE = 1900/2.52 ms, FA = 9°, thickness = 1.0 mm, slices = 192, matrix = 256 × 256, FOV = 250 mm).

Resting-state BOLD fMRI data were preprocessed using fMRIPrep (version 1.4.1) (Esteban et al., 2019). Briefly, the T1-weighted (T1w) image was corrected for intensity non-uniformity using N4BiasFieldCorrection (ANTs) and served as the T1w-reference throughout the workflow. Subsequently, a BOLD reference volume and its skull-stripped version were generated employing a custom method in fMRIprep. The BOLD reference was co-registered with the T1w reference utilizing bbregister from FreeSurfer. Head-motion parameters relative to the BOLD reference were estimated prior to any spatiotemporal filtering using mcflirt from FSL. After that, slice-time correction was applied to the BOLD runs utilizing 3dTshift from AFNI, followed by resampling into MNI152NLin2009cAsym standard volumetric space. Framewise Displacement (FD) was computed for each functional run (Power et al., 2012). Four subjects were excluded due to a mean FD > 0.3, resulting in 80 subjects for subsequent data analysis.

To account for potential confounding effects of physical and physiological noise, resting state fMRI data were further denoised using linear regression incorporating several nuisance regressors. These nuisance regressors included six realignment parameters and their temporal derivatives, as well as physiological noise estimated using the aCompCor method described by Behzadi et al. (Behzadi et al., 2007). The aCompCor method involved selecting the top five principal components from cerebrospinal fluid and white matter masks calculated in T1w space. Additionally, a first-order Legendre polynomial was included to address linear detrending. The residual time series were then subjected to temporal band-pass filtering, with a frequency range of 0.01–0.1 Hz.

### ROIs-based FC analyses

The MNI coordinates provided by Power et al. were used to construct ROIs (a sphere with a radius of 5 mm) of the DMN, FPN, and SN (Power et al., 2011). Specifically, the DMN consisted of 58 ROIs, while the FPN and SN comprised 25 and 18 ROIs respectively. The average time course was extracted from each ROI. To generate FC matrix, Pearson's correlation coefficients were computed for those averaged time course. Fisher's r-to-z transformation was applied for each correlation coefficient to fit the normal distribution using the rsHRF toolbox (Wu et al., 2021).

### FC-based predictive modeling

The NBS-Predict toolbox (www.nitrc.org/projects/nbspredict/), which integrates a machine learning model with connected graph components in a cross-validation framework (Serin et al., 2021), was employed to identify predictive model of ruminative reflection. Specifically, the NBS-Predict utilizes FC matrices and reflection scores to build a predictive model of reflection. To determine the edge features for model prediction on the training set, a general linear model with age, sex, and mean FD as covariates was applied. Initially, edges were selected based on a predefined threshold (p-value = 0.005), followed by identification of connected components within the set of suprathreshold edges. Subsequently, linear support vector regression, using the default parameter settings provided by NBS-predict without hyperparameter optimization, was trained on the suprathreshold edges from the largest connected component, and then applied to predict reflection scores in the test dataset. Finally, the weighted adjacency matrices of the connected components in all outer folds are averaged and scaled to obtain a mean weighted network. The weight value for each connection represents the presence of an edge in the selected connected component and reflects the model's prediction performance in each iteration of cross-validation, indicating the relative contribution of edges to the overall model (Serin et al., 2021).

The performance of the predictive model was evaluated using 10-repeated 10-fold cross-validation. Model performance assessment involved calculating the Pearson correlation coefficient (r) and root mean squared error (rMSE) between the predicted and actual reflection scores. To evaluate the statistical significance of these metrics, permutation tests were performed by randomly shuffling reflection scores 5000 times. The significance threshold was established at p<0.05, determined by the proportion of iterations in which the model trained on the permuted datasets exhibited performance similar to or better than the model trained on the original dataset.

## Results

### Behavioral results

The ruminative brooding and reflection scores were significant correlated with BDI scores (r = 0.67, p< 0.001; r = 0.38, p < 0.001) after controlling for age and sex. Brooding scores were positively correlated with reflection scores (r = 0.62, p < 0.001) after controlling for age, sex, and BDI-II scores.

### NBS-predict results

**Fig. 1:**
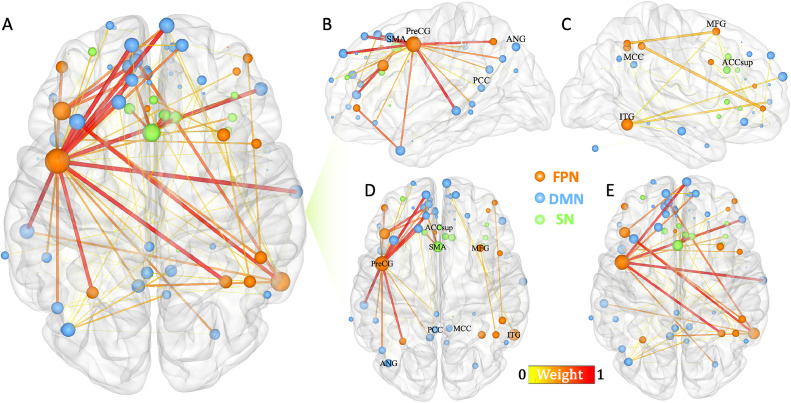
Visualization of the weighted subnetwork on a 3D brain surface associated with reflection score (A). The size and color of the nodes and edges are depicted based on the nodal degree and edge weight (no threshold applied; weight reflects the relative contribution of edges to the overall model). The weighted subnetwork was decomposed into left-hemispheric (B), right-hemispheric (C), intra-hemispheric (D), and inter-hemispheric connections (E). Abbreviation: ACCsup, anterior cingulate cortex-supracallosal; ANG, angular gyrus; ITG, Inferior temporal gyrus; MFG, middle frontal gyrus; MCC, middle cingulate - paracingulate gyri; PCC, posterior cingulate gyrus; PreCG, precentral gyrus; SMA, supplementary motor area.

**Fig. 2:**
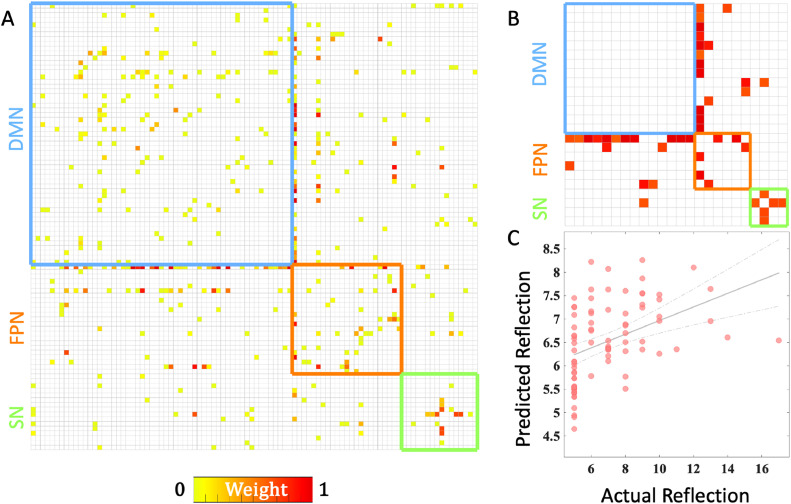
Weighted adjacency matrix showing connections associated with reflection score (A: no threshold applied; B: weight threshold = 0.6). (C) Scatter plot showing actual and predicted reflection.

The linear support vector regression model revealed that a subnetwork (268 links) comprising the DMN, FPN, and SN regions could predict individual ruminative reflection scores (Fig. 1). Specifically, FC within and between the DMN, FPN, and SN was found to predict reflection (Fig. 1), with detailed results for specific brain regions provided in supplementary Fig. S2. More importantly, connectivity within the FPN and SN, as well as between the FPN and DMN, contributed more significantly to the predictive model (Fig. 2A, B). The predicted reflection scores showed a significant correlation with actual reflection scores (r = 0.44, $p_{permutation}$= 0.002; rMSE = 2.23, $p_{permutation}$ = 0.011) (Fig. 2C). As the predefined threshold is inevitably arbitrary, we repeated the analyses with two alternative edge-selection thresholds (*p*-value = 0.001 and 0.01). Similar results were obtained: reflection was predicted by connectivity within and between the DMN, FPN, and SN (Fig. S3). The FPN and SN connectivity, as well as the FPN-DMN connectivity, had greater influence on the predictive model (Fig. S4A, B; S5A, B). The predicted reflection scores significantly correlated with the actual reflection scores at alternative thresholds *p* = 0.001 (*r* = 0.47, $p_{permutation}$ = 0.004; rMSE = 2.22, $p_{permutation}$ = 0.002; Fig. S4C) and *p* = 0.01 (*r* = 0.44, $p_{permutation}$ = 0.002; rMSE = 2.18, $p_{permutation}$ = 0.003; Fig. S5C).

## Discussion

In the present study, the NBS-predict approach revealed that FC within and between the DMN, FPN, and SN predicted individual levels of ruminative reflection. Notably, FC within the FPN and SN, as well as between the FPN and DMN, contributed more significantly to the predictive model. The prominent role of FPN and SN connectivity suggests that adaptive reflective processes are closely linked to enhanced executive control for internal emotional representation. The involvement of the well-established triple-network model, particularly the FPN-DMN coupling, underscores the importance of coordinating self-referential and goal-directed states in ruminative reflection.

We found that reflection was predicted by FC within the DMN and cognitive control-related networks (i.e., the FPN and SN). The DMN is known to support self-relevant thoughts, including autobiographical memory, self-referential thinking, and theory of mind (Buckner et al., 2008; Spreng et al., 2009). The prediction of reflective rumination by DMN connectivity demonstrates the involvement of self-referential activity in reflective processes (Luo et al., 2016; Satyshur et al., 2018). Compared to the DMN connectivity, the FPN and SN connectivity contribute more significantly to the predictive model. The FPN, primarily involved in cognitive control, is crucial for guiding flexible, goal-directed behavior (Seeley et al., 2007; Vincent et al., 2008). The SN, acting as a bottom-up processor of both external and internal salient events, initiates cognitive control by signaling the engagement of the FPN while suppressing DMN activity (Sridharan et al., 2008; Uddin, 2015). Previous studies suggest that FPN and SN functioning may influence the ability to exert cognitive control over ruminative thoughts and could determine the type of rumination (Lois & Wessa, 2016). Reflective rumination, characterized as an active problem-solving thought process, is associated with increased executive control, and serves as a coping strategy (Cristea et al., 2013; Stewart et al., 2019; Treynor et al., 2003). The recruitment of the FPN and SN is thus critical for purposeful, self-distancing, reflective processing. To date, only one study examined the relationship between reflection and FPN/SN connectivity in healthy subjects (Li et al., 2022). Li et al. (2022) found that reflection correlated with FC in the insula subregion and DLPFC. The greater contribution of FPN and SN connectivity in predicting reflection may provide empirical evidence that reflective rumination is an adaptive process and a potential strategy for regulating negative emotions (Cristea et al., 2013; Treynor et al., 2003).

Functional interactions between the DMN, FPN, and SN were found to predict reflection, suggesting a critical role of the triple-network model in reflection. As noted above, the SN is pivotal in coordinating DMN-related self-states and FPN-related goal-states (Menon & Uddin, 2010; Sridharan et al., 2008). Specifically, the SN can suppress DMN activity to promote disengagement from internally directed thoughts, thereby facilitating FPN activation for goal-directed behavior (Menon, 2011). Disruptions in SN coordination between the FPN and DMN have been implicated in depressive rumination (Guha et al., 2021; Hamilton et al., 2011; Pisner et al., 2019). Therefore, functional integration of the DMN, FPN, and SN is important for shifting from negatively biased self-referential processing to goal-directed thinking or planning. More importantly, the coupling between the FPN and DMN contributed more significantly to the predictive model. The FPN and DMN are highly integrated during task engagement and at rest (Fox et al., 2005). This integration is responsible for a wide range of executive functions, including cognitive flexibility, inhibitory control, and working memory (Cole et al., 2012; Dajani & Uddin, 2015; Douw et al., 2016). Previous studies have linked the FPN-DMN coupling with the ability to exert cognitive control over ruminative thinking (Lydon-Staley et al., 2019; Watkins, 2008). For example, repetitive negative thinking about self-related topics during rumination was related to inadequate regulation from the FPN to the DMN (Lydon-Staley et al., 2019). Taken together, our results support the involvement of the triple-network model in reflective processes and highlight the key role of FPN-DMN coupling in predicting reflection.

One potential limitation of this study is that our findings were derived from a cohort of healthy adults and may not generalize to individuals with clinical depression. While ruminative brooding and reflection were separate constructs in non-clinical groups, they became intertwined in clinical samples, thereby blurring the distinction between them (Joormann et al., 2006). Furthermore, although reflection may be adaptive in non-clinical populations, its adaptive properties can become conflated with the maladaptive properties of brooding in clinical populations (Stewart et al., 2019). Previous research has indicated that findings from the reflection subscale in nonclinical samples may not generalize to clinical populations (Whitmer & Gotlib, 2011). Further research is needed to confirm the role of reflection across both clinical and non-clinical populations.

## Conclusion

In this study, we found that reflective rumination in healthy subjects was predicted by connectivity within and between the DMN, FPN, and SN. Notably, the FPN and SN connectivity, as well as the FPN-DMN connectivity, made a greater contribution to the predictive model. The involvement of DMN connectivity indicates an association between reflection and self-referential processing. Relative to the DMN connectivity, the FPN and SN connectivity had a greater impact on the prediction of reflection, highlighting the critical role of cognitive control in reflective processes. Additionally, the well-established triple-network model, particularly the FPN-DMN coupling is implicated in reflection, suggesting that reflective processes involve the coordination of self-relevant and goal-directed mental activity. These findings advance our knowledge of the adaptive nature of reflection and may help inform the development of more targeted, effective interventions for preventing depression.

## Declaration of competing interest

The authors report no potential conflicts of interest.

## Appendix Supplementary materials

Image, application 1
