# Supplementary material for: Decoding ruminative reflection in healthy individuals: The role of triple network connectivity
Source: Int J Clin Health Psychol. 2024 Oct 10;24(4):100508. doi: 10.1016/j.ijchp.2024.100508 (PMC11735996; doi:10.1016/j.ijchp.2024.100508)
Supplement: Supplementary file 1 [file mmc1.docx]

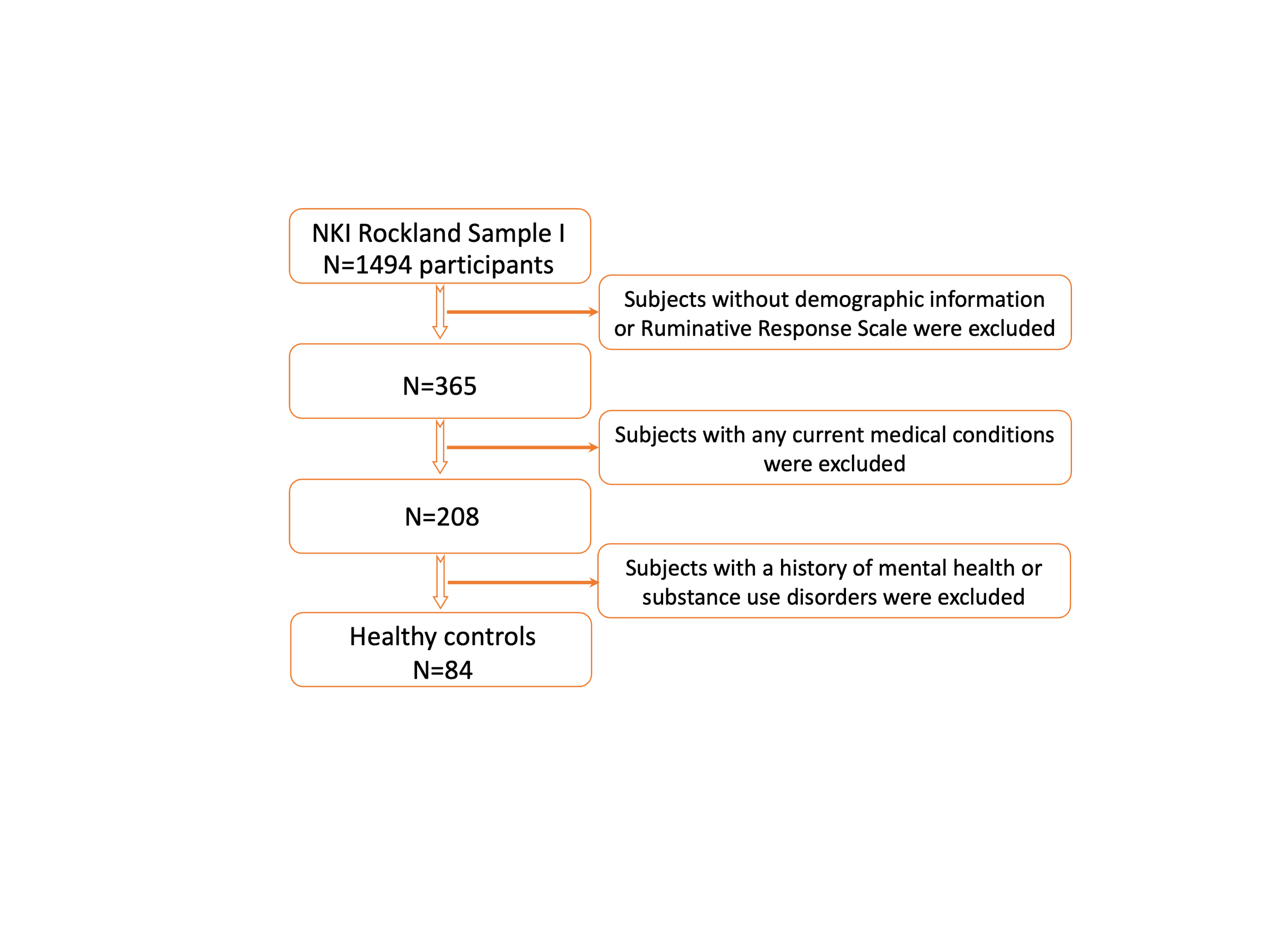


**Figure S1.** Flowchart of exclusion criteria and eligible subjects


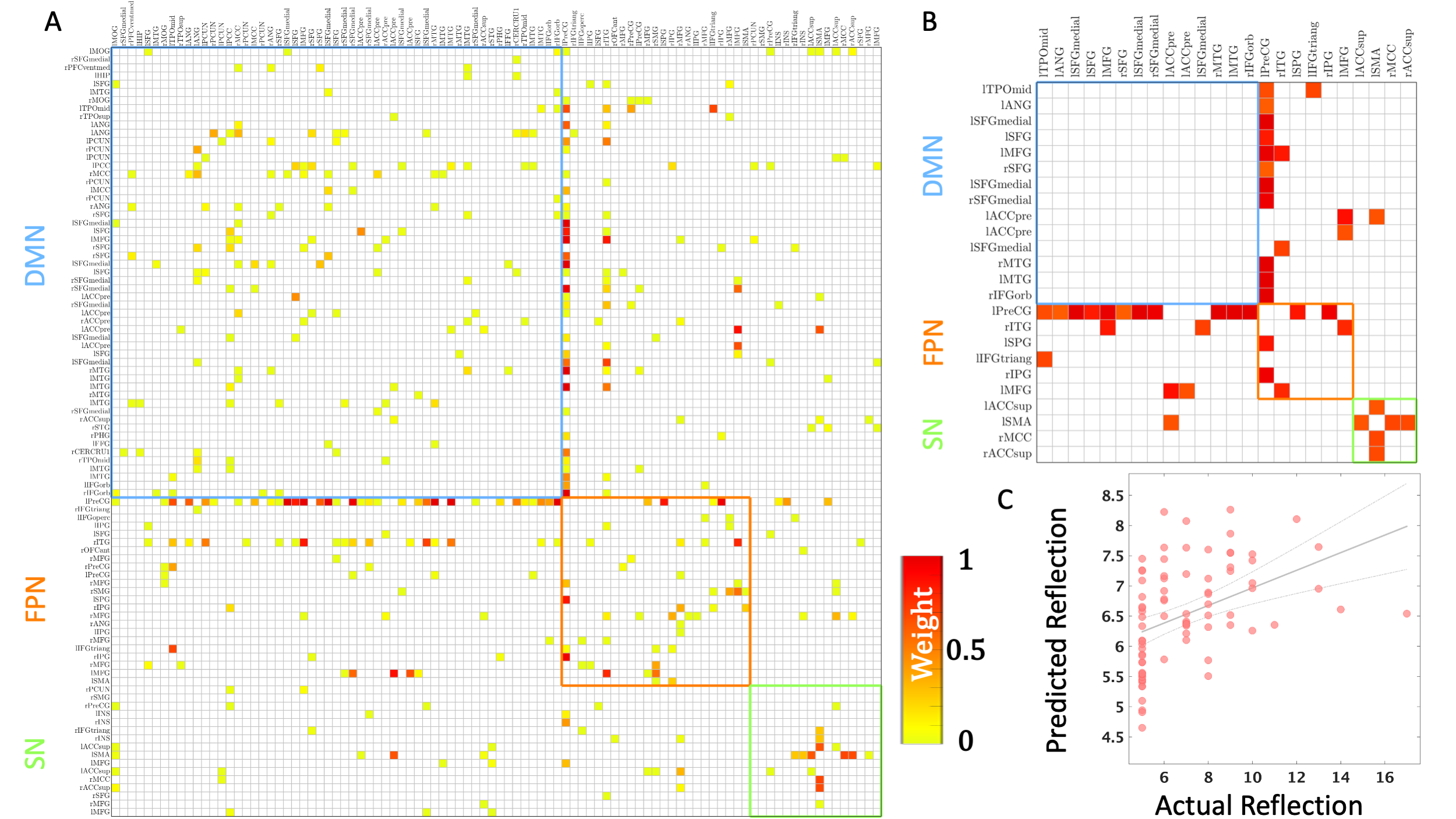


**Figure S2**. Weighted adjacency matrix showing connections associated with reflection score based on predefined threshold $p$-value = 0.005 (A: no threshold applied; B: weight threshold = 0.6). (C) Scatter plot showing actual and predicted reflection. The abbreviation for brain regions can be referenced in table S1.


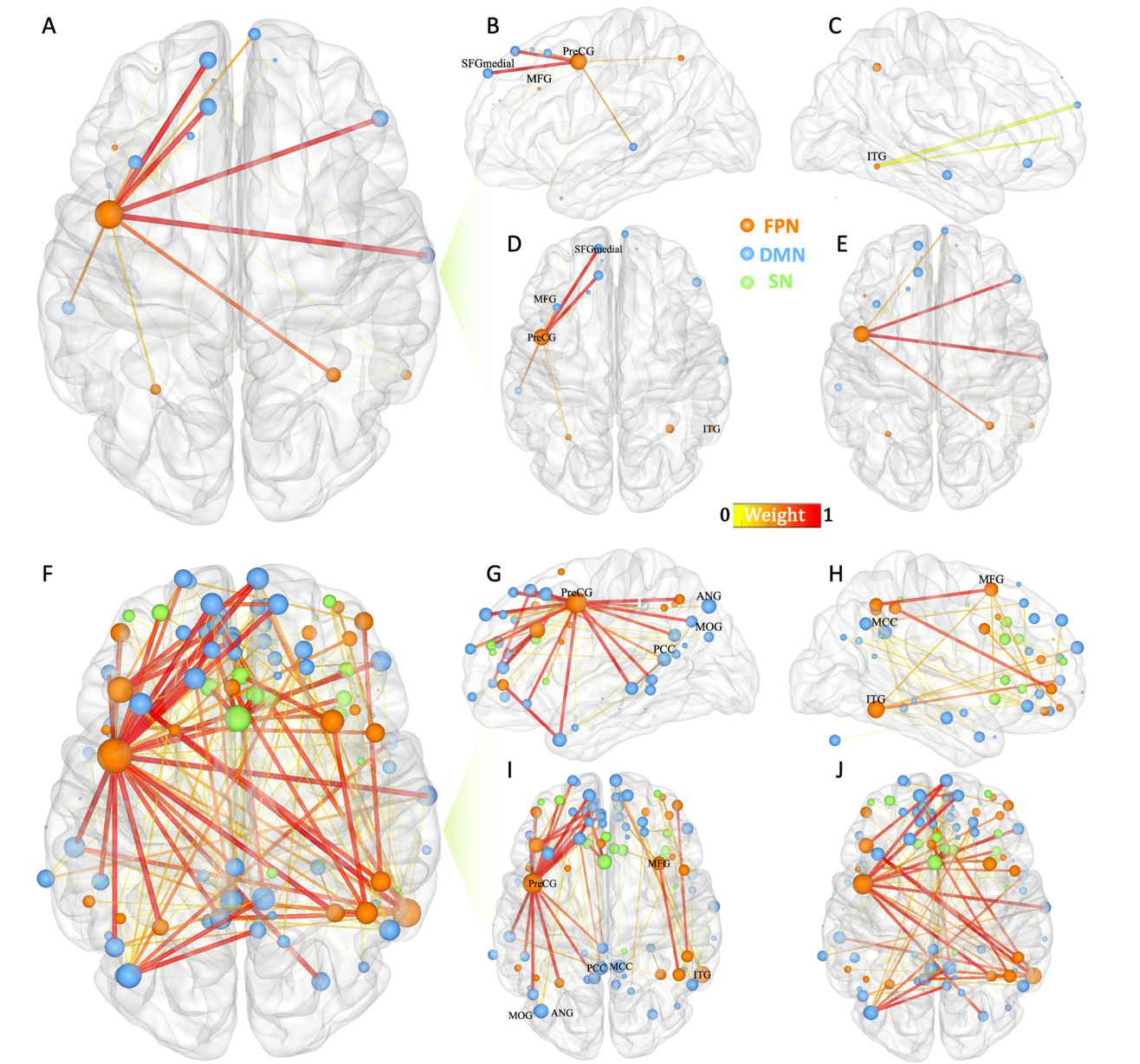


**Figure S3**. Visualization of the weighted subnetwork on a 3D brain surface associated with reflection score based on two different predefined thresholds (A: $p$ -value = 0.001, F: $p$ -value = 0.01). The size and color of the nodes and edges are depicted based on the nodal degree and edge weight (no threshold applied; weight reflects the relative contribution of edges to the overall model). The weighted subnetwork was decomposed into left-hemispheric (B & G), right-hemispheric (C & H), intra-hemispheric (D & I), and inter-hemispheric connections (E & J).


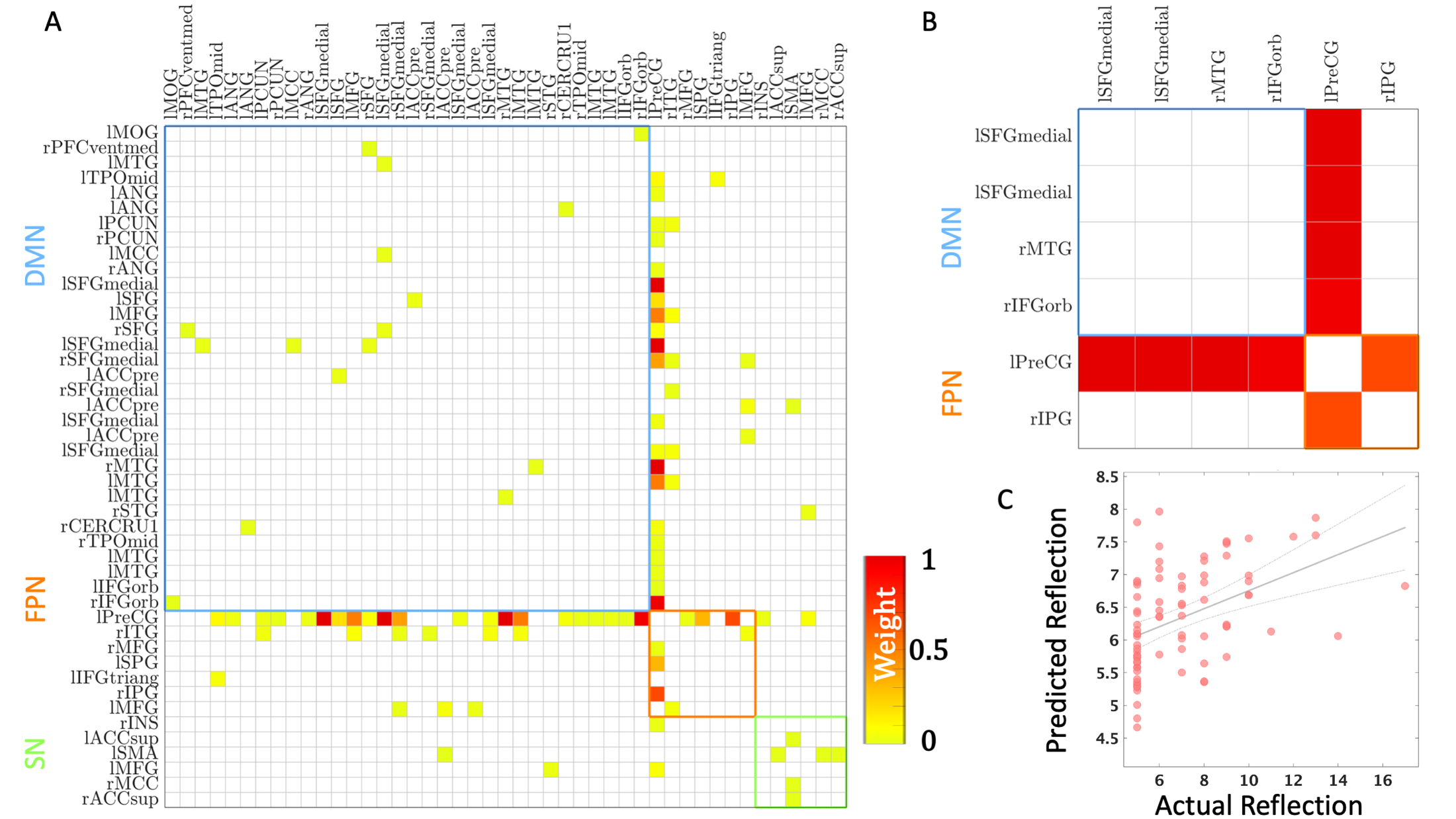


**Figure S4**. Weighted adjacency matrix showing connections associated with reflection score based on predefined threshold $p$ -value = 0.001 (A: no threshold applied; B: weight threshold = 0.6). (C) Scatter plot showing actual and predicted reflection. The abbreviation for brain regions can be referenced in table S1.


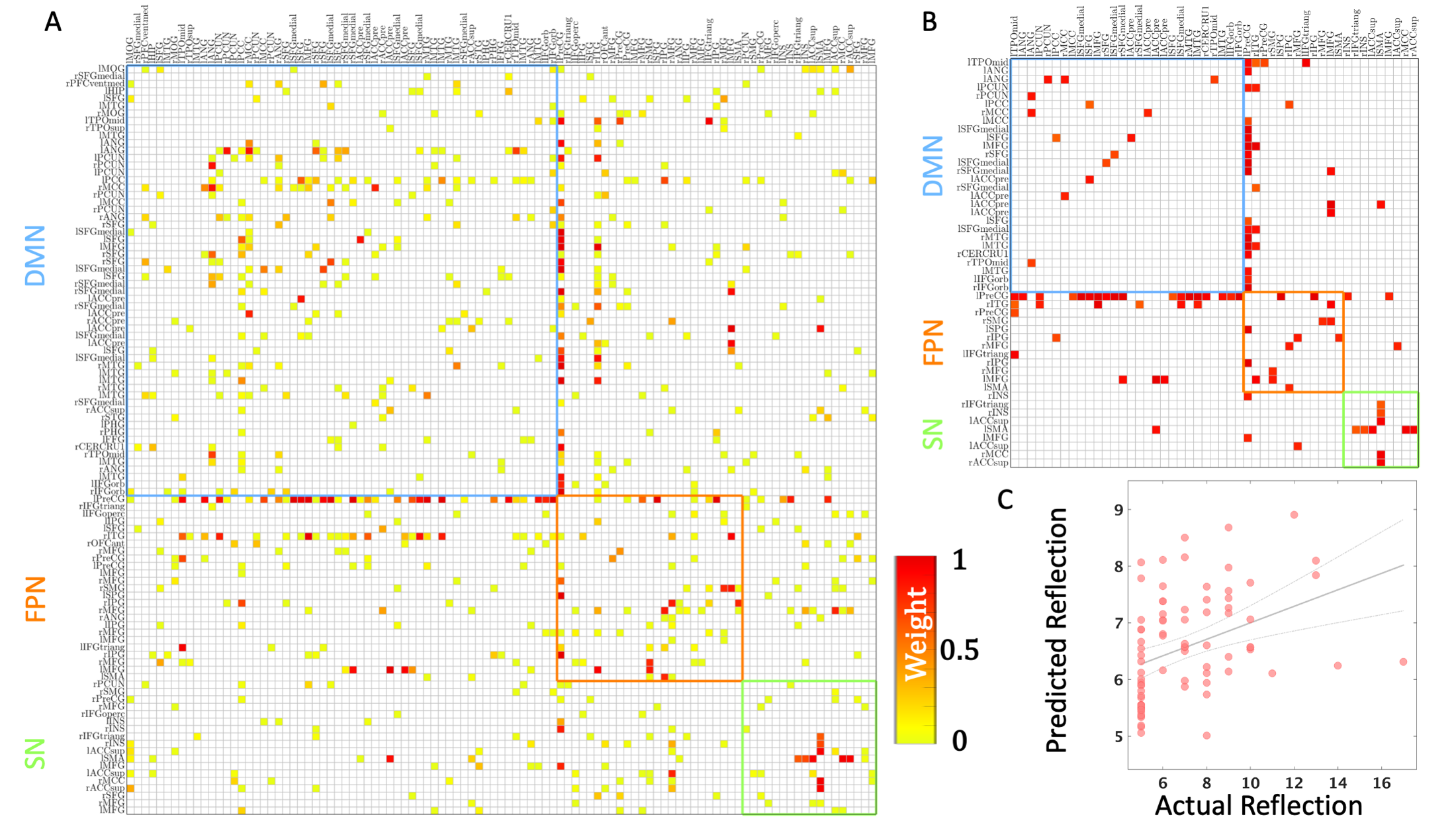


**Figure S5**. Weighted adjacency matrix showing connections associated with reflection score based on predefined threshold $p$ -value = 0.01 (A: no threshold applied; B: weight threshold = 0.6). (C) Scatter plot showing actual and predicted reflection. The abbreviation for brain regions can be referenced in table S1.

**Table S1. Coordinates in MNI space.**

| ROI# | x | y | z | network | AAL-Abbreviation | AAL |
| --- | --- | --- | --- | --- | --- | --- |
| 1 | 11 | -39 | 50 | Salience | rPCUN | Precuneus R |
| 2 | 55 | -45 | 37 | Salience | rSMG | SupraMarginal gyrus R |
| 3 | 42 | 0 | 47 | Salience | rPreCG | Precentral gyrus R |
| 4 | 31 | 33 | 26 | Salience | rMFG | Middle frontal gyrus R |
| 5 | 48 | 22 | 10 | Salience | rIFGoperc | Inferior frontal gyrus-opercular part R |
| 6 | -35 | 20 | 0 | Salience | lINS | Insula L |
| 7 | 36 | 22 | 3 | Salience | rINS | Insula R |
| 8 | 37 | 32 | -2 | Salience | rIFGtriang | Inferior frontal gyrus-triangular part R |
| 9 | 34 | 16 | -8 | Salience | rINS | Insula R |
| 10 | -11 | 26 | 25 | Salience | lACCsup | Anterior cingulate cortex-supracallosal L |
| 11 | -1 | 15 | 44 | Salience | lSMA | Supplementary motor area L |
| 12 | -28 | 52 | 21 | Salience | lMFG | Middle frontal gyrus L |
| 13 | 0 | 30 | 27 | Salience | lACCsup | Anterior cingulate cortex-supracallosal L |
| 14 | 5 | 23 | 37 | Salience | rMCC | Middle cingulate - paracingulate gyri R |
| 15 | 10 | 22 | 27 | Salience | rACCsup | Anterior cingulate cortex-supracallosal R |
| 16 | 31 | 56 | 14 | Salience | rSFG | Superior frontal gyrus-dorsolateral R |
| 17 | 26 | 50 | 27 | Salience | rMFG | Middle frontal gyrus R |
| 18 | -39 | 51 | 17 | Salience | lMFG | Middle frontal gyrus L |
| 19 | -44 | 2 | 46 | Frontoparietal | lPreCG | Precentral gyrus L |
| 20 | 48 | 25 | 27 | Frontoparietal | rIFGtriang | Inferior frontal gyrus-triangular part R |
| 21 | -47 | 11 | 23 | Frontoparietal | lIFGoperc | Inferior frontal gyrus-opercular part L |
| 22 | -53 | -49 | 43 | Frontoparietal | lIPG | Inferior parietal gyrus-excluding supramarginal and angular gyri L |
| 23 | -23 | 11 | 64 | Frontoparietal | lSFG | Superior frontal gyrus-dorsolateral L |
| 24 | 58 | -53 | -14 | Frontoparietal | rITG | Inferior temporal gyrus R |
| 25 | 24 | 45 | -15 | Frontoparietal | rOFCant | Anterior orbital gyrus R |
| 26 | 34 | 54 | -13 | Frontoparietal | rMFG | Middle frontal gyrus R |
| 27 | 47 | 10 | 33 | Frontoparietal | rPreCG | Precentral gyrus R |
| 28 | -41 | 6 | 33 | Frontoparietal | lPreCG | Precentral gyrus L |
| 29 | -42 | 38 | 21 | Frontoparietal | lMFG | Middle frontal gyrus L |
| 30 | 38 | 43 | 15 | Frontoparietal | rMFG | Middle frontal gyrus R |
| 31 | 49 | -42 | 45 | Frontoparietal | rSMG | SupraMarginal gyrus R |
| 32 | -28 | -58 | 48 | Frontoparietal | lSPG | Superior parietal gyrus L |
| 33 | 44 | -53 | 47 | Frontoparietal | rIPG | Inferior parietal gyrus-excluding supramarginal and angular gyri R |
| 34 | 32 | 14 | 56 | Frontoparietal | rMFG | Middle frontal gyrus R |
| 35 | 37 | -65 | 40 | Frontoparietal | rANG | Angular gyrus R |
| 36 | -42 | -55 | 45 | Frontoparietal | lIPG | Inferior parietal gyrus-excluding supramarginal and angular gyri L |
| 37 | 40 | 18 | 40 | Frontoparietal | rMFG | Middle frontal gyrus R |
| 38 | -34 | 55 | 4 | Frontoparietal | lMFG | Middle frontal gyrus L |
| 39 | -42 | 45 | -2 | Frontoparietal | lIFGtriang | Inferior frontal gyrus-triangular part L |
| 40 | 33 | -53 | 44 | Frontoparietal | rIPG | Inferior parietal gyrus-excluding supramarginal and angular gyri R |
| 41 | 43 | 49 | -2 | Frontoparietal | rMFG | Middle frontal gyrus R |
| 42 | -42 | 25 | 30 | Frontoparietal | lMFG | Middle frontal gyrus L |
| 43 | -3 | 26 | 44 | Frontoparietal | lSMA | Supplementary motor area L |
| 44 | -41 | -75 | 26 | Default Mode | lMOG | Middle occipital gyrus L |
| 45 | 6 | 67 | -4 | Default Mode | rSFGmedial | Superior frontal gyrus-medial R |
| 46 | 8 | 48 | -15 | Default Mode | rPFCventmed | Superior frontal gyrus-medial orbital R |
| 47 | -13 | -40 | 1 | Default Mode | lHIP | Hippocampus L |
| 48 | -18 | 63 | -9 | Default Mode | lSFG | Superior frontal gyrus-dorsolateral L |
| 49 | -46 | -61 | 21 | Default Mode | lMTG | Middle temporal gyrus L |
| 50 | 43 | -72 | 28 | Default Mode | rMOG | Middle occipital gyrus R |
| 51 | -44 | 12 | -34 | Default Mode | lTPOmid | Temporal pole-middle temporal gyrus L |
| 52 | 46 | 16 | -30 | Default Mode | rTPOsup | Temporal pole-superior temporal gyrus R |
| 53 | -68 | -23 | -16 | Default Mode | lMTG | Middle temporal gyrus L |
| 54 | -44 | -65 | 35 | Default Mode | lANG | Angular gyrus L |
| 55 | -39 | -75 | 44 | Default Mode | lANG | Angular gyrus L |
| 56 | -7 | -55 | 27 | Default Mode | lPCUN | Precuneus L |
| 57 | 6 | -59 | 35 | Default Mode | rPCUN | Precuneus R |
| 58 | -11 | -56 | 16 | Default Mode | lPCUN | Precuneus L |
| 59 | -3 | -49 | 13 | Default Mode | lPCC | Posterior cingulate gyrus L |
| 60 | 8 | -48 | 31 | Default Mode | rMCC | Middle cingulate - paracingulate gyri R |
| 61 | 15 | -63 | 26 | Default Mode | rPCUN | Precuneus R |
| 62 | -2 | -37 | 44 | Default Mode | lMCC | Middle cingulate - paracingulate gyri L |
| 63 | 11 | -54 | 17 | Default Mode | rPCUN | Precuneus R |
| 64 | 52 | -59 | 36 | Default Mode | rANG | Angular gyrus R |
| 65 | 23 | 33 | 48 | Default Mode | rSFG | Superior frontal gyrus-dorsolateral R |
| 66 | -10 | 39 | 52 | Default Mode | lSFGmedial | Superior frontal gyrus-medial L |
| 67 | -16 | 29 | 53 | Default Mode | lSFG | Superior frontal gyrus-dorsolateral L |
| 68 | -35 | 20 | 51 | Default Mode | lMFG | Middle frontal gyrus L |
| 69 | 22 | 39 | 39 | Default Mode | rSFG | Superior frontal gyrus-dorsolateral R |
| 70 | 13 | 55 | 38 | Default Mode | rSFG | Superior frontal gyrus-dorsolateral R |
| 71 | -10 | 55 | 39 | Default Mode | lSFGmedial | Superior frontal gyrus-medial L |
| 72 | -20 | 45 | 39 | Default Mode | lSFG | Superior frontal gyrus-dorsolateral L |
| 73 | 6 | 54 | 16 | Default Mode | rSFGmedial | Superior frontal gyrus-medial R |
| 74 | 6 | 64 | 22 | Default Mode | rSFGmedial | Superior frontal gyrus-medial R |
| 75 | -7 | 51 | -1 | Default Mode | lACCpre | Anterior cingulate cortex-pregenual L |
| 76 | 9 | 54 | 3 | Default Mode | rSFGmedial | Superior frontal gyrus-medial R |
| 77 | -3 | 44 | -9 | Default Mode | lACCpre | Anterior cingulate cortex-pregenual L |
| 78 | 8 | 42 | -5 | Default Mode | rACCpre | Anterior cingulate cortex-pregenual R |
| 79 | -11 | 45 | 8 | Default Mode | lACCpre | Anterior cingulate cortex-pregenual L |
| 80 | -2 | 38 | 36 | Default Mode | lSFGmedial | Superior frontal gyrus-medial L |
| 81 | -3 | 42 | 16 | Default Mode | lACCpre | Anterior cingulate cortex-pregenual L |
| 82 | -20 | 64 | 19 | Default Mode | lSFG | Superior frontal gyrus-dorsolateral L |
| 83 | -8 | 48 | 23 | Default Mode | lSFGmedial | Superior frontal gyrus-medial L |
| 84 | 65 | -12 | -19 | Default Mode | rMTG | Middle temporal gyrus R |
| 85 | -56 | -13 | -10 | Default Mode | lMTG | Middle temporal gyrus L |
| 86 | -58 | -30 | -4 | Default Mode | lMTG | Middle temporal gyrus L |
| 87 | 65 | -31 | -9 | Default Mode | rMTG | Middle temporal gyrus R |
| 88 | -68 | -41 | -5 | Default Mode | lMTG | Middle temporal gyrus L |
| 89 | 13 | 30 | 59 | Default Mode | rSFGmedial | Superior frontal gyrus-medial R |
| 90 | 12 | 36 | 20 | Default Mode | rACCsup | Anterior cingulate cortex-supracallosal R |
| 91 | 52 | -2 | -16 | Default Mode | rSTG | Superior temporal gyrus R |
| 92 | -26 | -40 | -8 | Default Mode | lPHG | Parahippocampal gyrus L |
| 93 | 27 | -37 | -13 | Default Mode | rPHG | Parahippocampal gyrus R |
| 94 | -34 | -38 | -16 | Default Mode | lFFG | Fusiform gyrus L |
| 95 | 28 | -77 | -32 | Default Mode | rCERCRU1 | Crus I of cerebellar hemisphere R |
| 96 | 52 | 7 | -30 | Default Mode | rTPOmid | Temporal pole-middle temporal gyrus R |
| 97 | -53 | 3 | -27 | Default Mode | lMTG | Middle temporal gyrus L |
| 98 | 47 | -50 | 29 | Default Mode | rANG | Angular gyrus R |
| 99 | -49 | -42 | 1 | Default Mode | lMTG | Middle temporal gyrus L |
| 100 | -46 | 31 | -13 | Default Mode | lIFGorb | IFG pars orbitalis L |
| 101 | 49 | 35 | -12 | Default Mode | rIFGorb | IFG pars orbitalis R |
